# Hepatitis E Virus RNA Detection in Liver and Muscle Tissues Sampled from Home Slaughtered Domestic Pigs in Central Italy

**DOI:** 10.1007/s12560-024-09606-2

**Published:** 2024-06-11

**Authors:** Gianluigi Ferri, Giorgia Giantomassi, Daniele Tognetti, Alberto Olivastri, Alberto Vergara

**Affiliations:** 1 Department of Veterinary Medicine, Specialization School in Food Inspection “G. Tiecco”, University of Teramo, Piano d’Accio, Strada Porvinciale 18, 64100 Teramo, Italy; 2 Ascoli Piceno, Italy; 3 SIAOA Veterinary Public Service, Ascoli Piceno, Italy

**Keywords:** Hepatitis E virus, Liver and muscle, Environmental sharing, Molecular biology, Food safety, One-health

## Abstract

Hepatitis E virus is a worldwide emerging foodborne pathogen; raw or undercooked meats and liver pork products can cause infection through the orofecal route. In Central-Southern Italy, small traditional farming method, associated with the possibility of environmental sharing with wild species, can facilitate HEV diffusion and persistence. The aim of this study was to determine HEV genotype and subtype in Marche region from home slaughtered domestic pigs involved in small and traditional food chains. A total of 236 liver and muscle tissues and 6 pooled salami samples were screened. Laboratory workflow started with homogenization, followed by RNA extraction. Nested reverse transcription PCR and qRT-PCR were used to amplify specific parts of overlapping open reading frames belonging to the HEV genome. A total of 42/236 (17.79%) liver and 8/236 (3.39%) diaphragm specimens were positive; none of the pooled salami specimens showed positive HEV signal. The discovered HEV3c presented high nucleotide similarities with ones amplified from wild boar populations hunted in the same province. Extensive farming methods and environmental sharing with wild animal species support cross-infection infections, as observed in the present study. Although salami resulted negative for HEV RNA detection, the effects of food technologies on viral loads remain unclear. Therefore, further scientific investigations coupled with efficacious standardized laboratory procedures will be the next challenge.

## Introduction

Hepatitis E virus (HEV) is one of the most interesting foodborne pathogens investigated in food sciences, due to its environmental persistence and zoonotic infectious characteristics (Purdy et al., 2017). Basing on the viral life cycle, infections can occur after the ingestion of contaminated raw or under-cooked food matrices of animal origin (domestic swine as main reservoir) in the industrialized countries or also from water (domestic swine as main reservoir) in the incoming ones (Smith et al., 2020). Nowadays, thermal treatment (71 °C for 20 min) results as scientifically confirmed food process that can determine a consistent HEV inactivation (Barnaud et al., 2012).

HEV is a quasi-enveloped single strand RNA virus which is taxonomically classified in the *Hepeviridae* Family, Subfamily *Orthohepeviridae* belonging to the species *Paslahepevirus balayani*. Its structural characteristics permit to survive in different environments (Purdy et al., 2022). HEV genome is characterized by three overlapping Open Reding Frames (ORF): ORF1 encodes for nonstructural proteins, ORF2 for capsid protein and ORF3 for a small protein (Purdy et al., 2022). Among the eight identified HEV genotypes, HEV-3, whole or partial genome detection (which causes sporadic hospitalization cases in immune-competent human consumers), has been largely discovered from human sera (Aspinall et al., 2017), pig foodstuffs (Ahmed & Nasheri, 2023; Johne et al., 2022), etc. in the European and Northern American continents. More in detail, the epidemiological scenario highlighted a wide distribution of the above-mentioned genotype in many sample types collected from domestic pigs (feces, fresh liver and muscle tissues, and processed products) i.e., in Germany (Priemer et al., 2022), Italy (Carella et al., 2023), Netherlands (Hogema et al., 2021), Sweden (Wang et al., 2019), Slovakia (Jackova et al., 2021). Other two aspects which cover key-roles concerning HEV (including genotype 3) diffusion and persistence are: the environmental sharing with wild animal species [i.e., wild boars (*Sus scrofa*) and wild ruminants] associated with the traditional farming methods which are parts of small food production chains. In many regions, located in Central and Southern Italy (including Marche region), pig rearing at family level is very frequent. Animals are then slaughtered at home and their meat used for proven domestic consumption (De Sabato et al., 2022). Short supply chains and the lack of adequate health and hygiene controls, at different levels from slaughter to the consumption of fresh and processed meat, pose important questions about effective safety of these products. Extensive or semi-extensive farming methods can provide ideal conditions (as environmental sharing) where HEV could easily cross-infect domestic and wild reservoirs (i.e., wild boars and domestic pigs) (Aprea et al., 2018). Indeed, the scientific hypothesis of potential cross-species infections have been largely demonstrated in many recent studies (Aprea et al., 2018; Arnaboldi et al., 2021; Uema et al., 2022). Starting from these last affirmations, this study aimed to discover HEV RNA using biomolecular methods (nested reverse transcription PCR and qRT-PCR) with special regard to specific overlapping open reading frames (ORF): ORF1, ORF2, and ORF3. The following sequencing process was performed for the characterization of the detected viral genotypes. These methods resulted applied to evaluate the possible circulation of HEV RNA in a traditional and uninvestigated food production chain which starts from the home farming to the domestic processed meats i.e., salami. Consumption of home-made fermented and seasoned muscle tissues (salami) can pose at risk consumer’s health because the cooking process (known as the main virucidal effect) is not involved (Barnaud et al., 2012). For this purpose, a total of 236 animals (*Sus scrofa domesticus*) were involved. The target sampled organs were liver and muscle tissues collected from home slaughtered subjects, and following this small traditional food producing chain, six pooled salami specimens were also included. All screened subjects came from familial farms in Marche region (Central Italy) having high possibilities of environmental sharing with wild animal species [i.e., wild boars (*Sus scrofa*)]. Describing the local zootechnic characteristics, it is also mandatory to affirm that there is a low pig farm density classified as intensive production realities. The local cultural aspects, typical of the Ascoli Piceno province, are strictly connected with rooted traditions of *Suidae* Family (domestic and wild species) food production and consumption. It is also important to mention that home production usually mix domestic pig and wild boar meats for producing salami or dry sausages; this choice is related to many organoleptic and traditional aspects. In previous studies performed in the same province (Ferri et al., 2022a, 2022b), wild boars resulted positive to the HEV RNA detection. Therefore, this study was designed to fill a crucial scientific aspect as further molecular investigation applied to a small integrated food chain from fresh to processed pork foodstuffs. Basing on the previous results (observed from wild boars), the present studies tried also to emphasize the scientific hypothesis of possible cross-infections between the wild and urban viral life cycles that in many ecological conditions could influence each other, with possible sanitary repercussions on consumers.

## Materials and Methods

### Screened Population and Area

A population of 236 domestic pigs (*Sus scrofa domesticus*) was involved in the present scientific investigation. The screened population was composed by mixed-bred subjects, with an average slaughtering age between nine to twelve months; more detailed information are illustrated in the following Table 1.

**Table 1:** Animal population screened in the present study

Screened population	Average weight	Animal genders
236 domestic pigs	200 ± 20 kg	179/236 or 75.84% F (CI 95%: 70.38–81.30%)
		57/236 or 24.15% M (CI 95%: 18.69–29.61%)

*F* females, *M* males

**Fig. 1:**
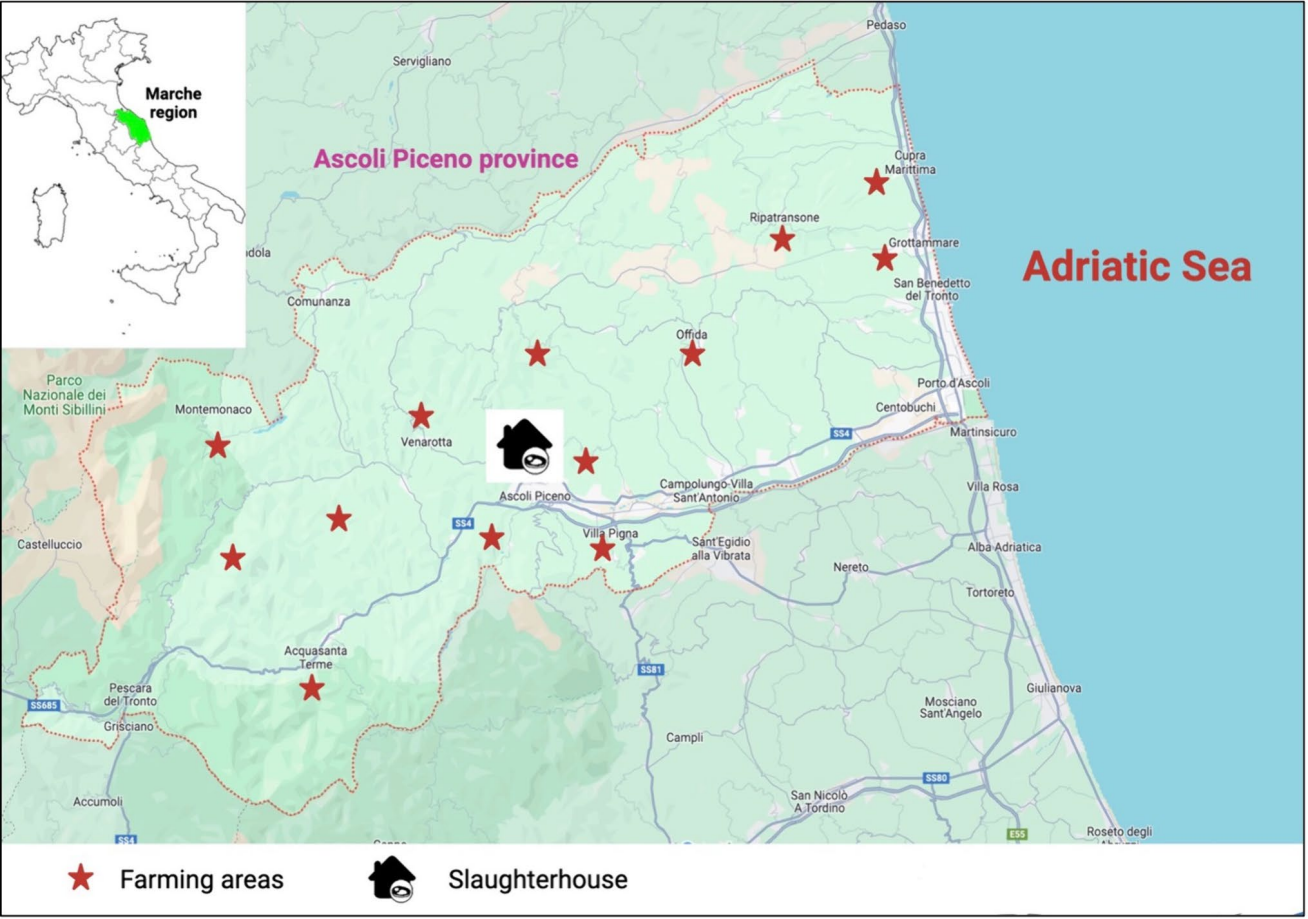
Geographical distribution of positive domestic pigs and their respective farming areas in the Ascoli Piceno province, Marche region, Italy

These animals were traditionally bred for familial consumption that it is generally represented by two subjects per farm following the extensive method. It means that animals live in paddocks which are alternate to free-range periods. The screened pigs came from a total of 112 domestic farms (1 or two bred subjects/farm) that were in the Ascoli Piceno province (Marche region, Italy). This area is characterized by wide rural geographical realities and it is also part of the Monti Sibillini National Park characterized by a total land surface of 71,437.00 ha covering different provinces belonging to the Umbria and Marche regions (See Fig. 1). Marche Regional report on wild species estimated the fauna demographic density (Regional Marche Hunting Report, 2020), with special regard to the wild boar species, 2.5/100 ha and for wild ruminants < 1/100 ha. *Sus scrofa* densities have increased due to the notable demographic growth associated with the lack of consistent populations of wild predators and due to the continuous expanding of the urbanization process.

### Samples Collection and Processing

The sample collections started as following step to the home slaughtering procedures. These ones were performed by specific law-designed professional figures, in agreement with the EU Reg. No. 625/2017 and No. 1069/2009. From each home slaughtered domestic pig, the respective pluck was transported under refrigerated conditions and conferred for the official sanitary *post-mortem* evaluation performed by the Veterinary Public Service at Ascoli Piceno slaughterhouse. At this step of the present investigation, two tissue aliquots of 15 ± 2 g: one from liver and the second one from diaphragm tissue were collected. The tissues separation method was aseptically performed. Liver and muscle specimens were sterilely sampled, using mono-usage scalpels (Swann-Morton®, Sheffield, UK), paying attention to the possible cross-contamination from serosa to the parenchyma, as described by Dzierzon et al., (2022). Samples were transported under refrigerated conditions to the laboratory (Department of Veterinary Medicine, University of Teramo, Italy) and were finally stored at −80 °C till the performance of qualitative and quantitative molecular assays. The specimen collection was characterized by a second step that involved the screened food chain. It included 6 pooled meat salami which were realized, by the domestic producers, using meats coming from 5 animals for each product (representing a final amount of 30 subjects). The information, about the screened subjects, were stored for traceability purposes; it permitted to link liver, muscle and salami specimens to the specific animals used for food production. Concerning salami production, other ingredients such as pepper and salt were also added to the ground meat. The following step was food aging period was 1 month. At the end of the last-mentioned step, the processes products were transported under refrigerated conditions from the domestic producers to the laboratory. At this level, aliquots of 15 ± 2 g of salami, in agreement with collections performed for liver and muscle tissues, were collected and stored at -80 °C till the molecular screenings.

After collection, the laboratory workflow was equally applied for all sample types following three different moments: homogenization, RNA extraction and molecular assays. Starting from the collected 15 g/aliquot/tissue type (liver, diaphragm, and salami ones), 5 g/tissue type were sterilely sectioned and homogenized using the T18 digital Ultra-Turrax® (IKA®-Werke GmbH & Co. KG, Staufen, Germany) adding 5 mL of phosphate buffered saline (PBS) solution (pH 7.4) (ThermoFisher™, Waltham, MA, USA). From each homogenate, samples were centrifugated at 4,000 rpm for 15 min at refrigerated temperature; after this last step, 500 µL of supernatant were collected from liver and muscle tissues and used for the RNA extraction protocol. Except for salami specimens, at the end of the homogenization process (performed like for liver and muscle tissues), the fatty supernatant layer was removed. Proteins and RNA precipitations were obtained using 200 µL of HCl solution 1 M (pH 3.5–4.0) and centrifugated at 4000 rpm for 15 min (4 °C). After supernatant removal, the obtained pellets were suspended in 5 mL of PBS solution pH 7.4 (ThermoFisher™, Waltham, MA, USA), as previously described by Dziedzinska et al., (2020). For all matrices, the RNA extraction process started from 500 µL aliquots and were performed following the TRIzol LS method (Invitrogen, Ltd., Paisley, UK) (Rio et al., 2010) that permitted to obtaining final pellets suspended in 50 µL of RNase free water (Invitrogen UltraPure DNase/RNase-Free Distilled Water, ThermoFisher™, Waltham, MA, USA) and including negative controls, as performed Wang et al., (2021). All extracts were finally stored at −80 °C.

### Quantitative Molecular Assays: RT-qPCR

The molecular quantitative analyses were the real time RT-qPCR assays. The first step included the standard curves constructions useful for quantification. The plasmid synthesis (cloning technique into pUC57), performed by BioFab Research Laboratories (Rome, Italy), permitted to obtain a specific and well-known amplicons concentration referring to the sequence position corresponding to 5261–5330 related to the accession number M73218 obtained from GenBank, as reported by Jothikumar et al. (2006). The second step was to perform tenfold dilutions, of the above-mentioned amplicons, starting from a volume of 2 µL of DNA [containing 4 × 10^9^ genome equivalent copies/g (GE/g)]. It permitted to generate curves from 10^9^ to 10^0^ GE/g of plasmid DNA, in agreement with procedures described by Jothikumar et al. (2006). Specific crossing points, calculated using the RAPID system software (Idaho Technology Inc., Salt Lake City, UT), provided the expected quantitative data as products of the real time RT-qPCR. These curves were constructed using the Real-time GENE UP® System (bioMérieux, Paris, France) to estimate the HEV GE/g values.

This step was followed by the RT-qPCR assays which were performed using the QuantiTec Probe RT-PCR kit (Qiagen®, Hilden, Germany) with final reaction volumes of 20 µL. In each reaction tube, 2 µL of extracted RNA was added. The ATCC® VR-3258SD RNA fragment was used as HEV positive control, and negative one was also included in each performed real time RT-qPCR assays. The exogenous internal positive control resulted added to each sample in order to supervise the appearance of potential PCR inihibitors. The used probe, as schematically represented in Table 1, was introduced with a specific concentration of 50 µM. The JVHEVF and JVHEVR primers are designed to anneal within a conserved region of the ORF-3 among the HEV-1 to HEV-4 genotypes. In addition, the used probe JVHEVP contained 5′ 6-carboxy fluorescein fluorophore and 3' black hole quencher, as indicated by Jothikumar et al. (2006). A schematic representation of the used couples of primers is illustrated in Table 1. Thermocycler settings start with the reverse transcription at 50 °C for 20 min and finish with extension at 40 °C for 30 s, as described by Barnaud et al. (2012). Negative controls were included in all assays.

### Nested RT-PCR and Gel Electrophoresis Analysis

All specimen types (liver and muscle tissues and salami) were also screened performing nested RT-PCR assays using the thermocycler Mastercycler® nexus X2–PCR Thermal Cycler (Eppendorf, Hamburg, Germany). The first reaction (reverse transcription) was the RT-PCR working with final reaction volumes of 25 µL using specific kits: OneStep RT-PCR kit (Qiagen®, Hilden, Germany) which included 5 µL of extracted RNA from each sample type. The second one (nested PCR) was performed in a final volume of 20 µL (Green Master Mix Promega®, Madison, WI, USA) adding 2.5 µL of products obtained from the first reaction. The used oligo primer couples targeted specific nucleotide regions belonging to HEV ORF1 and ORF2. In both reactions, negative and positive controls were also included. Thermocycler settings and the relative electrophoresis assays were performed in accordance with the respective references [ORF-1 Johne et al., (2010)] and [ORF-2 Wang et al., (1999)], as reported in the Table 2.

**Table 2:** Primers used for HEV RNA detection: qualitative and quantitative screenings

Reactions	Referring genes	Primers	Oligonucleotide sequences	Amplicon sizes	References
Real time RT-qPCR	ORF3	JVHEVF	GGTGGTTTCT GGGGTGAC	68 bp	Jothikumar et al., (2006)
		JVHEVR	AGGGGTTGGTTGGATGAA		
		JVHEVP	TGATTCTCAGCCCTTCGC		
RT-PCR	ORF2	ConsORF2-s1	GACAGAATTRATTTCGTCGGCTGG	197 bp	Wang et al., (1999)
		ConsORF2-a1	CTTGTTCRTGYTGGTTRTCATAATC		
Nested PCR		ConsORF2-s2	GTYGTCTCRGCCAATGGCGAGC	145 bp	
		ConsORF2-a2	GTTCRTGYTGGTTRTCATAATCCTG		
RT-PCR	ORF1	HEV-cs	TCGCGCATCACMTTYTTCCARAA	470 bp	Johne et al., (2010)
		HEV-cas	GCCATGTTCCAGACDGTRTTCCA		
Nested PCR		HEV-csn	TGTGCTCTGTTGGCCCNTGGTTYG	333 bp	
		HEV-casn	CCAGGCTCACCRGARTGYTTCTTCCA		

*Bp* base pairs, *F* forward, *R* reverse, *P* probe

Nested PCR products were successively loaded onto agarose gels at different concentrations i.e., 1.5% or 2.0% depending on the amplicon sizes. Among wells, two types of specific DNA ladders were used where each standard line corresponded to 50 bp or 100 bp (^©^NIPPON Genetics EUROPE, Düren, Germany). Negative and positive controls were also loaded. The running set was 80 Volts for 40 min. The suspected positive bands, compared to the standard bands, were successively purified and sequenced, as described in the following paragraph.

### Sanger Sequencing

The expected positive bands, to the electrophoresis analysis steps, were purified using the Qiagen QIAquick® PCR Purification Kit (Hilden, Germany), and successively sequenced through the Sanger method by BioFab Research Laboratories (Rome, Italy). An initial sequences evaluation was performed using the BLASTN system (https://blast.ncbi.nlm.nih.gov/Blast.cgi, accessed on 03 June 2023). The HEV genotype and subtype identification were also studied with the HEV NET typing tool (https://www.rivm.nl/mpf/typingtool/hev/), as previously indicated by Mulder et al. (2019). The nucleotide similarities, alignments and the evolutionary analysis were specifically studied and performed using the MEGA X Software, as previously demonstrated by Kumar et al. (2018). The Neighboring-Joining method was used to construct the phylogenetic trees, as suggested by Saitou and Nei (1987). The obtained positive sequences were successively deposited and published in the GenBank database (https://www.ncbi.nlm.nih.gov/genbank/). The deposited sequences were successively published (on 11 December 2023). The referring sequences were registered with the following accession numbers: OR933697 and OR933698 for ORF1 fragment (333 bp) on the GenBank platform, as above indicated. Basing on the phylogenetic analysis, high nucleotide similarities with other sequences are described the Results section.

### Statistical Analysis

Concerning the qualitative data, the chi-square non-parametric statistical value was defined using the IBM® SPSS Statistics [Version:29.0.0.0 (241)] and results were considered statistically significant if *p*-values were < 0.05. The real time RT-qPCR results were analyzed calculating the *t* test to compare differences in the screened population correlating geographical areas to the animals’ gender. Results were considered statistically significant when *p*-value was < 0.05. The confidential intervals (CI) at 95% were calculated for all provided percentages.

## Results

From November 2022 to January 2023, a total of 236 home slaughtered domestic swine (*Sus scrofa domesticus*) fresh matrices i.e., liver and muscle tissues, and processed ones i.e., salami, farmed in the Ascoli Piceno province (Monti Sibillini National Park), were molecularly investigated for the HEV RNA detection. Forty-two out of two hundred and thirty-six liver 17.79% (CI 95%: 12.92–22.66%) and 8/236 diaphragm tissues (3.39% CI 95%: 1.09–5.69%) were positive for HEV RNA detection amplifying specific genomic regions belonging to the ORF-1 (nested RT-PCR assays), and ORF-3 (real time ones) genes. Animals with positive diaphragm tissue presented HEV RNA in their respective hepatic one (to the nested RT-PCR ORF-1 fragments detections). More in detail, among positive liver tissues, the gender variable showed that 32/42 (76.19% CI 95%: 63.31–89.07%) of positive subjects were female, and 10/42 (23.81% CI 95%: 10.93–36.69%) males. The obtained positive diaphragms were 5/8 (62.50% CI 95%: 28.96–96.04%) from female and 3/8 (37.50% CI 95%: 3.96–71.04%) from male subjects, as illustrated in the Table 3.

**Table 3:** Positive sample results according to the gender, tissues and localization variables

Sample types	Positive LV	LV and gender	Positive DG	DG and gender	Positive S
236 LV	42/236	32 F	8/236	5/8 F	0 F
236 DG		10 M		3/8 M	0 M
6 S (30 subjects)					

N. Positive LV and gender	N. Positive DG and gender	Geographical localization
6F*; 3 M	2F*	AP 42.51 N; 13.34 E
5F; 1 M	2F*	Roccafluvione 42.51 N; 13.28 E
1F; 1 M*	1 M*	Folignano 42.49 N; 13.38 E
3F	–	Venarotta 42.53 N; 13.29 E
2F	–	Offida 42.56 N; 13.42 E
1F	–	Grottammare 42.59 N; 13.52 E
1F; 2 M	–	Rotella 42.57 N; 13.33 E
3F	1 M	Ripatransone 42.59 N; 13.45 E
2F	–	Castignano 42.56 N; 13.37 E
1F; 1 M*	1 M*	Cupra Marittima 43.1 N; 13.51 E
2F; 1 M	–	Acquaviva Picena 42.56 N; 13.48 E
2F*; 1 M	1F*	Massignano 43.3 N; 13.47 E
3F	–	Appignano 43.21 N; 13.20 E

*LV* Liver, *DG* diaphragm, *F* female, *M* male, *AP* Ascoli Piceno districts

*Subjects positive to the HEV RNA detection both from DG and LV tissues

None of screened salami samples, analyzed performing real-time qPCR (ORF-3) and nested RT-PCR (ORF-1 and ORF-2) assays, resulted positive. Among the eight positive diaphragm samples, six animals were also used by the familiar producers, in the pooled salami processes. Based on the geographical distributions of the different screened familial husbandries, it was observed that positive subjects, were mostly farmed and home slaughtered in Ascoli Piceno and small rural subareas were also included as part of the territorial legal competence located 6 km from the city center i.e., Roccafluvione, Rotella and Ripatransone districts. Complete and detailed data are illustrated in the following Fig. 1 and Table 3.

The used TaqMan analysis determined the amount of a specific genome fragment, belonging to the HEV ORF-3 gene, which was observed from all 42 positive animals. The observed quantitative assays were also followed by a qualitative confirmation performing the nested RT-PCR ones. Positive liver samples mostly detected 10^2^ GE/g, excepting for one subject (farmed in Venarotta) that presented 10^3^ GE/g. The obtained Cq values were included between 28.3 and 35.1 cycles with a mean value of 31.7 in liver tissues. Concerning the diaphragm tissues, positive animals presented 10^2^ GE/g characterized by Cq values between 30.4 and 37.5 with a mean of 33.9.

**Fig. 2:**
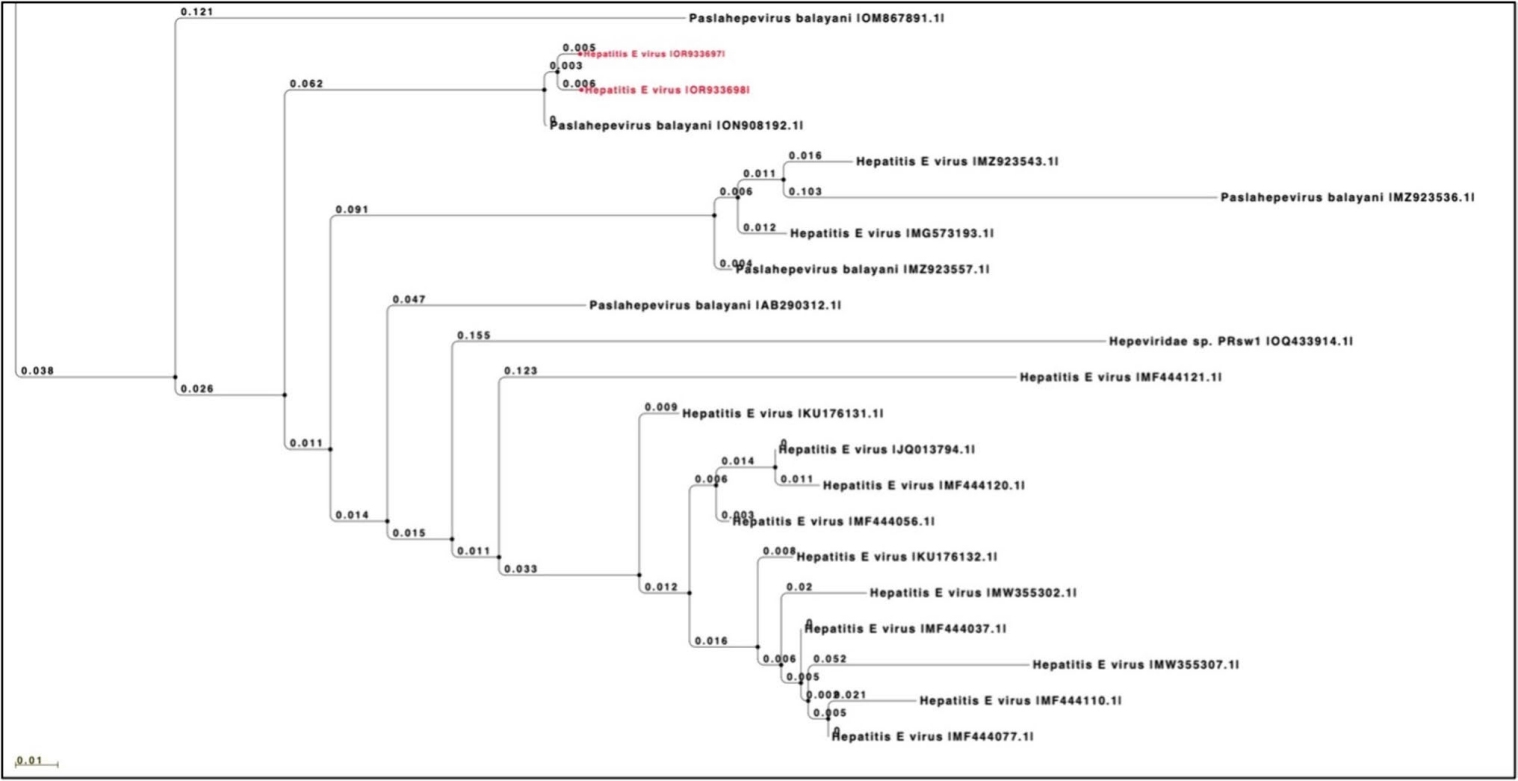
Neighboring-joining phylogenetic tree constructed basing on the nucleotide fragment of 333 bp, referring to HEV ORF1 from liver and muscle samples. The p-distance model with bootstrapping of 1000 replicates was performed. Sequences OR933697 and OR933698 (red marked) were discovered from liver and muscle tissue

The registered sequences (accession numbers: OR933697 and OR933698 for ORF1 fragment 333 bp) were loaded and the obtained data from the BLASTN alignment and the HEV NET typing tools showed 100.0% of nucleotide identity to the HEV3 subtype c (firstly confirmed by the phylogenetic analysis) that was discovered from wild boars (*Sus scrofa*) livers and diaphragms in the Ascoli Piceno province (the same screened area of this scientific investigation) one year ago (Ferri et al., 2022b): GenBank accession number: ON908192.1. The registered sequences were selected basing on the proper nucleotide similarities with the ones published on the GenBank platform, as graphically illustrated by the phylogenetic tree in the Fig. 2.

### Statistical Analysis

Starting from qualitative results, obtained from the nested RT-PCR assays, the applied non-parametric test (chi-square value with Yate’s correction) highlighted a first statistical difference comparing the positive liver tissues between female and male subjects (*χ*^2^: 21.735 with a *p*-value < 0.001) to the ORF-1 targeting amplicon detections. A similar result was also observed for positive diaphragm tissues analyzed basing on the gender variable reporting a final *χ*^2^: 18.262 and *p*-value was < 0.002. Positive female subjects presented a statistically significant difference if compared with the male ones (*p*-value < 0.001).

## Discussion

In Marche region, as observed in other Italian ones (Costanzo et al., 2015; Ianiro et al., 2021; Pavia et al., 2021), there is a small and rooted domestic swine (*Sus scrofa domesticus*) farming traditional method (classified as semi-extensive). It is characterized by small pigs’ numbers/farm (generally 2 pigs/family) located in National Park (Monti Sibillini National Park), and this farms still use domestic pig manures for the typical vegetable home-gardens. Generally, the fresh and processed pork products (i.e., salami), obtained from these animals, are mainly ingested by consumers who live in the same province. For this reason, this small food production chains could have sanitary repercussions in a well-defined geographical area and cannot enter in the industrial commercial circuit, in agreement with the EU Reg. No. 853/2004 and No. 625/2017. The obtained matrices can represent possible HEV sources for the human infection.

In this study, performed in the Ascoli Piceno province, HEV RNA was amplified from 42/236 pigs representing 17.79% (CI 95%: 12.92–22.66%) of the screened animals. The obtained prevalence value resulted quite higher if compared with other similar studies; as observed in Sicily region, where scientists discovered HEV RNA in 21/210 or 17.50% (CI 95% 12.36–22.64%) animals (Lorusso et al., 2023). Our data was also higher than ones reported by Uema et al., (2022) in Japan who amplified part of the viral genome from 20/200 or 10.00% pigs (CI 95% 4.29–13.27%) and by García et al. (2020) from 7/45 or 15.55% (CI 95% 4.97–26.13) pig liver samples. More in detail, all positive pigs, belonging to the present scientific investigation, presented HEV RNA sequences from their respective liver tissues; while 8/42 (9.52% CI 95% 0.65–18.39%) of positive subjects also detected HEV RNA in the diaphragm tissues too. These results were in line with other studies performed in Germany 8.14% of muscle tissues (Priemer et al., 2022), and higher than the data discovered in Spain 4.31% (García et al., 2020). In this study none of the screened animals resulted positive for HEV RNA detection to the muscle only; indeed, positive diaphragms were also positive for HEV RNA in the respective liver tissue. Indeed, HEV RNA was never discovered only from muscle tissues (obtained from liver positive pigs) resulting in line with other previous investigations in the same animal species (García et al., 2020; Lorusso et al., 2023; Uema et al., 2022). From a comparative perspective, in the *Suidae* Family, wild boars (*Sus scrofa*) also presented the same observations, as suggested by many authors (De Sabato et al., 2022; Forzan et al., 2021; Lo Presti et al., 2020). It is also important to mention that the present scientific investigation focuses on traditional small food chain. This variable must be considered in comparison with intensive farming method and to the industrial food processing. These findings get their scientific explanation relating to the fact that hepatocytes represent the target cell for HEV replication, and gall bladder is involved as viral microenvironment for storage. Muscular tissues are mainly colonized during the viremic phase of the infection status (Oechslin et al., 2022). In this study, possible cross-contaminations during samples collections cannot be considered because high sterile procedures were applied. Indeed, HEV migrations, during cuttings from serosa to the parenchyma, were prevented, as indicated by Dzierzon et al. (2022), considering the high risks due to the anatomical contiguity between liver and diaphragm.

The discovered GE/g (10^2^–10^3^ GE/g from livers and 10^2^ GE/g from diaphragm tissues) in this study, amplified performing the RT-qPCR, indicate low risks for immunocompetent consumers. However, it could represent a public health concern for immune-compromised subjects (Purdy et al., 2017). The discovered HEV GE/g in liver and muscle tissues were in line with other analogous studies (De Sabato et al., 2022; Forzan et al., 2021; Lorusso et al., 2023).

From an ecological perspective, the environmental sharing between domestic and wild species can provide strategical epidemiological conditions for HEV cross-species infection and its persistence in specific geographical areas i.e., National Parks (Arnaboldi et al., 2021; Bonardi et al., 2020). In this study, all animals were farmed in specific rural realities located in the Monti Sibillini National Park and Ascoli Piceno province represents a part of it. Due to the high wild boar densities (as described in the “Material and Methods” section), that also resulted positive to the HEV RNA detection in previous studies performed in the same province (Ferri et al., 2022a, 2022b), the scientific hypothesis, that possible cross-species infections occurred, could find explanation by the high nucleotide similarities that emerged during the phylogenetic analysis, as illustrated in the Fig. 2 (“Results”). This last affirmation was demonstrated by the nucleotide alignments (100% nt. identity) between ones discovered from pigs (in this study, GenBank accession numbers: OR933697 and OR933698) and sequences discovered one year ago from wild boars (*Sus scrofa*) published as ON908192.1 by Ferri et al. (2022b). Wild ruminants were excluded as possible HEV reservoirs because of the low density in the Ascoli Piceno province, as previously mentioned in the “Material and Methods” section.

The obtained results in the processed products as salami did not amplify oligonucleotide fragments belonging to: ORF-1, ORF-2 (nested RT-PCR), and ORF-3 (real time qPCR). This finding resulted in line with other similar Italian studies (Montone et al., 2019; Moro et al., 2021). It is mandatory to consider that salting and seasoning processes, that in this case lasted of 1 month, probably determined the HEV RNA sequences denaturation (Moro et al., 2021), which were amplified from fresh products, but not detected from the same muscle tissues (with special regard to the diaphragm one) used for salami productions. Analogous results were observed by other scientific investigations; however, potential virucidal effects of aging are not well studied, as reported by Wolff et al., (2020) and Locus et al. (2023). More specifically, Wolff et al. (2020) observed that HEV was resistant to extreme pH values ranged between 2 and 9. These last-mentioned pH values exclude most of animal origin food matrices which are typically characterized by an average value of pH: 5 (i.e., pork salami products). This critical aspect confirms that the cooking process (considering the initial viral loads) remains the unique virucidal food processing treatment (Barnaud et al., 2012). The absence of HEV RNA in the screened salami, belonging to the present study and obtained from muscle-positive animals, could be related to the HEV sequences GE/g low concentration associated with the consistent dilutions that could be occurred. However, this first preliminary results, obtained from the processed pork products should be furtherly investigated. The possible correlations between initial viral loads and food processes will represent consistent future challenges.

Based on a gender point of view, female subjects 32/42 (76.19% CI-95%: 63.31–89.07%) were more positive than male ones 10/42 (23.81% CI-95%: 10.93–36.69%), as described in the Results section. The *t*-test, concerning the RT-qPCR assay results, showed a significant statistical difference (*p*-value < 0.001) comparing the observed GE/g of genomic sequences amplified from liver and muscle samples. Gender influence, as hormone-dependent biochemical system, was supposed by many studies in the *Suidae* Family (Aprea et al., 2018; Arnaboldi et al., 2021; Forzan et al., 2021). For these reasons, the obtained results could be justified by the scientific hypothesis that female hormones can act as triggers determining more receptive hosts to the viral replication. Indeed, this last affirmation found its scientific fundamentals in a specific study performed by Singh et al. (2019).

Therefore, this paper proposes interesting data concerning HEV RNA circulation, and more specifically HEV-3c, in small and traditional farms of domestic pigs. Animals are home slaughtered according to procedures (in agreement with the European Legislations) and their meats used for self-consumption in small food production chains. The increasing relevance of viral pathogens, involved in many infectious outbreaks, should cover a crucial role to stimulate the scientific community and the European institutions to include viral foodborne pathogens (i.e., HEV, norovirus GI and GII, hepatitis A virus, etc.) in the so-called food safety criteria, described in the EU Reg. No. 2073/2005. These legislative implementations result mandatory due to the wide diversity of animal origin productions, and the regional guidelines do not result enough to manage and limit viruses. Therefore, consumers and environmental health will be ensured involving the biomolecular screenings as surveillance methods as medical preventive tools. Surveillance of zoonotic foodborne pathogens, based on well-structured sanitary laws covering from artisanal to industrial level, will contribute to the reduction of infections.

## Conclusions

The so called One-health approach was applied in this biomolecular investigation. Starting from veterinary medicine (that included epidemiological results) and continuing with the human one (repercussions on the consumer health), the environmental medicine involves a crucial role. The possible cross-species infections, among wild and domestic environments, find their scientific exp53lanation from HEV structural characteristics. This resistance behavior significantly influences the epidemiological variable. Therefore, basing on the obtained scientific evidences, the present study highlights as farming methods and environmental sharing can provide crucial possibilities for HEV particles persistence. Concerning food processes, the traditional method, for salami production, still lack of specific data on the efficacy of food aging on HEV virions. For this reason, there is a scientific necessity to fill this gap with further investigations. From a futuristic point of view, the physical parameters (i.e., high hydrostatic pressures, ultrasounds, etc.) and nanoparticles involvement will be able to provide fascinating effects on the viral structures.

## Data Availability

No datasets were generated or analysed during the current study.
